# Activating transcription factor 3: A potential therapeutic target for inflammatory pulmonary diseases

**DOI:** 10.1002/iid3.1028

**Published:** 2023-09-22

**Authors:** Dandan Li, Juanjuan Jing, Xue Dong, Chenyang Zhang, Jia Wang, Xianyao Wan

**Affiliations:** ^1^ Department of Critical Care Medicine The First Affiliated Hospital of Dalian Medical University Dalian China

**Keywords:** acute lung injury, acute respiratory distress syndrome, ATF3, chronic obstructive pulmonary disease, posttranslational modifications, pulmonary fibrosis

## Abstract

**Background:**

Activating transcription factor 3 (ATF3) is a nuclear protein that is widely expressed in a variety of cells. It is a stress‐inducible transcription gene and a member of the activating transcription factor/cAMP responsive element‐binding protein (ATF/CREB) family.

**Methods:**

The comprehensive literature review was conducted by searching PubMed and Google Scholar. Search terms used were “ATF3”, “ATF3 and (ALI or ARDS)”, “ATF3 and COPD”, “ATF3 and PF”, and “ATF3 and Posttranslational modifications”.

**Results:**

Recent studies have shown that ATF3 plays a critical role in many inflammatory pulmonary diseases, including acute lung injury (ALI)/acute respiratory distress syndrome (ARDS), chronic obstructive pulmonary disease (COPD), and pulmonary fibrosis (PF). ATF3 participates in many signaling pathways and complex pathophysiological processes, such as inflammation, immunity, endoplasmic reticulum stress, and cell proliferation. However, the role of ATF3 in current studies is controversial, and there are reports showing that ATF3 plays different roles in different pulmonary diseases.

**Conclusions:**

In this review, we first summarized the structure, function, and mechanism of ATF3 in various inflammatory pulmonary diseases. The impact of ATF3 on disease pathogenesis and the clinical implications was particularly focused on, with an overall aim to identify new targets for treating inflammatory pulmonary diseases.

## Graphical abstract

In this review, we first summarize the structure, function, and signaling pathways of ATF3, and then introduce the mechanism of ATF3 in various inflammatory pulmonary diseases. We focus on the impact of ATF3 on disease pathogenesis and clinical implications, aiming to provide new targets for treating inflammatory pulmonary diseases.

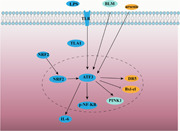

## 1 INTRODUCTION

During respiration, the lungs are constantly exposed to various irritants, such as bacteria, viruses, cigarette smoke, and airborne particulates, which makes them highly susceptible to pulmonary diseases. The characteristics of pulmonary diseases include high rates of morbidity and mortality, as well as poor treatment outcomes, making them a significant public health problem worldwide.^1^ In studies on the pathogenesis of pulmonary diseases, it has been found that transcription factors directly affect gene expression and play a vital role in the regulation of cellular functions and disease development, and consequently, these have thus become a hot topic of research in recent years.^2^, ^3^

Activating transcription factor 3 (ATF3) is a stress‐inducible transcription factor that belongs to the activating transcription factor/cAMP‐responsive element‐binding protein (ATF/CREB) family, which regulates gene transcription by forming homodimers or heterodimers via the basic leucine zipper (bZIP) structural domain, thereby supporting the biological functions of genes.^4^, ^5^, ^6^ ATF3 mainly functions as an adaptive response gene to maintain genetic integrity and intracellular homeostasis under stress conditions.^7^

The expression of ATF3 is relatively stable under normal physiological conditions, while changes in its expression are associated with multiple pathophysiological responses (e.g., inflammation, oxidative stress, endoplasmic reticulum stress, and cell death).^8^, ^9^, ^10^ This review briefly discusses the mechanism of action of ATF3 and its effects on biological functions and cellular processes, with a special focus on the role of ATF3 in acute lung injury (ALI)/acute respiratory distress syndrome (ARDS), chronic obstructive pulmonary disease (COPD), and pulmonary fibrosis (PF), and with an overall aim to identify new targets for the treatment of inflammatory pulmonary diseases.

## 2 STRUCTURE AND BIOLOGICAL FUNCTIONS OF ATF3

### 2.1 Structure of ATF3

**Figure 1:**
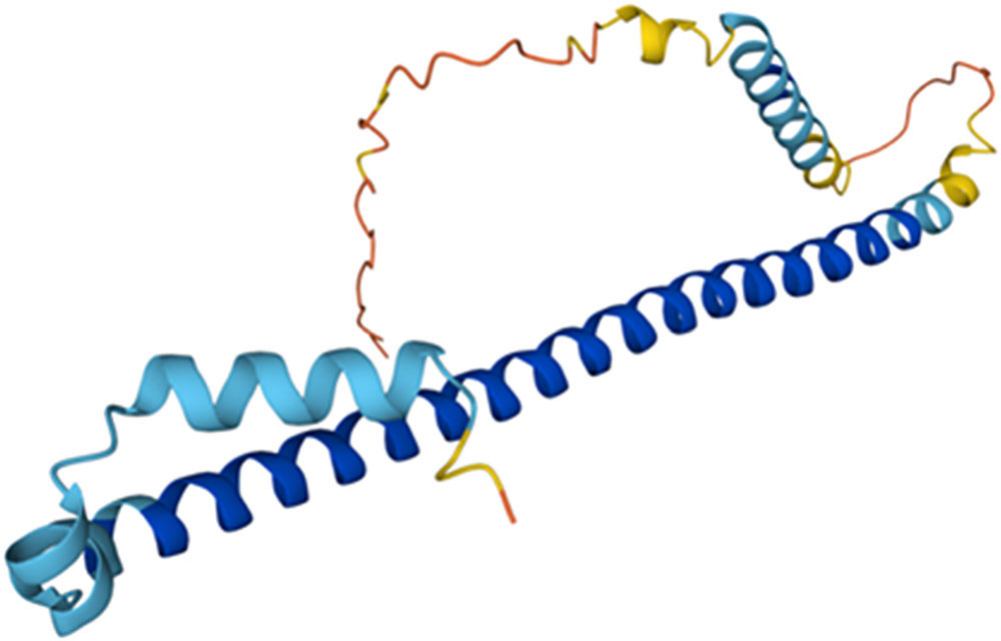
The spatial structure of ATF3.

ATF3 was first identified from a cDNA library of HeLa cells stimulated by serum. ATF3 contains four exons and encodes 181 amino acids (Figure 1). The molecular weight of ATF3 is 22 kDa.^11^ ATF3 has the same binding site as other ATF/CREB family transcription factors, which is 5′‐TGACGTCA‐3′.^12^ Among the same family, the more studied ones are ATF1, ATF2, ATF4, and CREB.^13^ They interact with target DNA by binding an entire region in the bZIP structural domain.

### 2.2 Biological functions of ATF3

#### 2.2.1 Transcriptional regulation of ATF3

The stimulation mode and cell type mainly determine the function of ATF3. Although the ATF3 gene is expressed at low levels in normal conditions, its expression is upregulated when subjected to multiple stimuli, such as hypoxia, cytokines, DNA damage, or chemotherapeutic drugs.^14^, ^15^ The ATF3 promoter can bind to the loci involved in cellular stress responses to accomplish the transcriptional regulation of these genes.^16^ Thus, ATF3 acts as a regulator in the host defense mechanism.^17^

ATF3 is a star gene of the ATF/CREB family and its dimerization affects gene transcription. ATF3 can not only act with other ATF/CREB family members, such as JDP2, but also with other family transcription factors, such as p53, Nrf2, and nuclear factor‐κB (NF‐κB), to regulate gene expression.^18^, ^19^, ^20^, ^21^ ATF3 binds to regulatory sequences within the promoter region of a target gene, which can lead to its transactivation or repression.^16^, ^22^, ^23^ The specific mechanisms by which ATF3 regulates transcription remain to be further explored.

#### 2.2.2 Posttranslational modifications of ATF3

**Figure 2:**
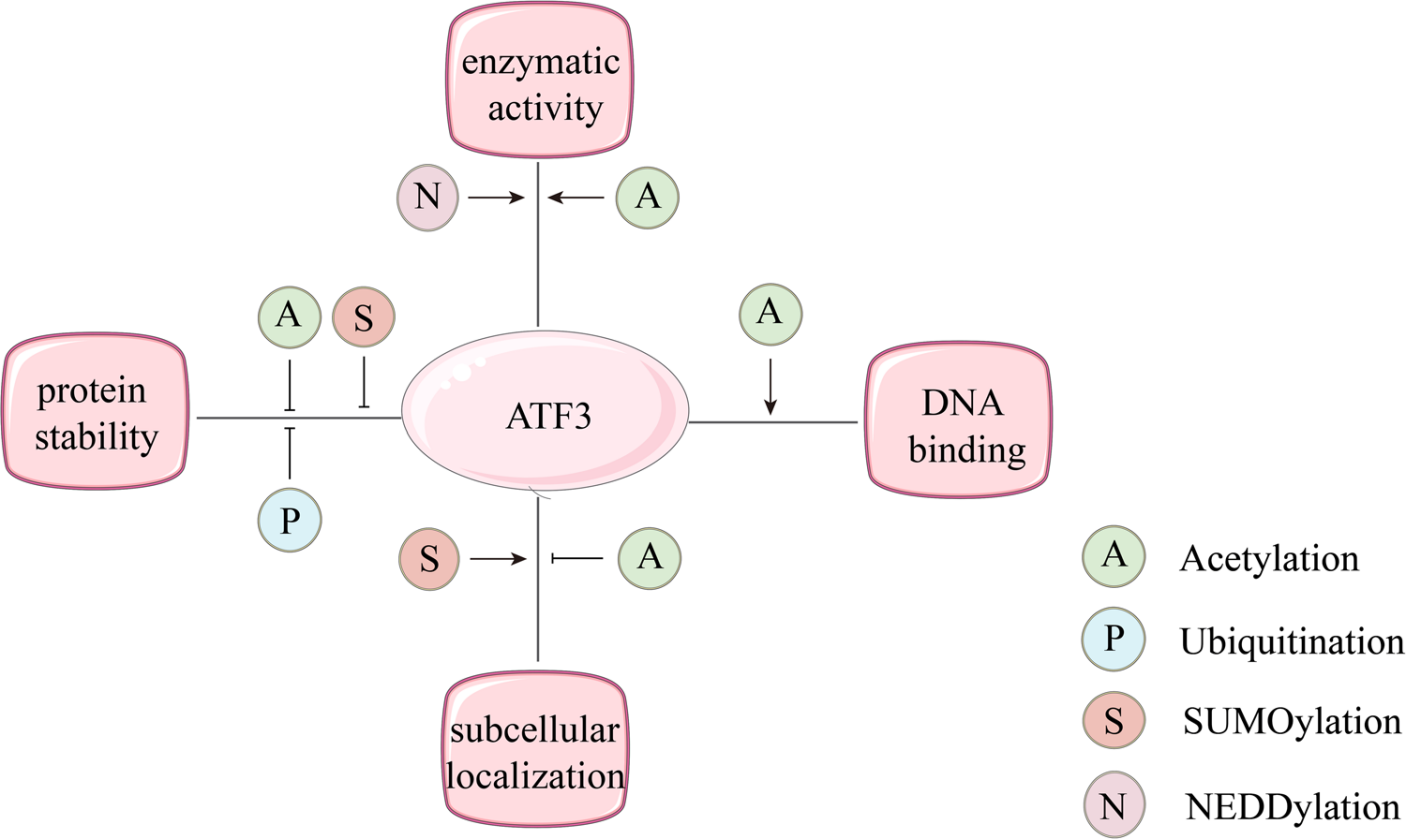
Posttranslational modification plays a critical role in regulating ATF3 enzymatic activity, DNA binding, protein stability, and subcellular localization.

ATF3 can be modified by acetylation, ubiquitination, and ubiquitin‐like modification, and these posttranslational modifications change its stability and function (Figure 2).

##### Acetylation

Protein acetylation modifications are involved in several important physiological functions, such as transcriptional regulation, signaling pathways, metabolic regulation, protein stability, and responses to microbial infection.^24^, ^25^, ^26^ The mammalian intracellular acetyltransferases that mediate protein acetylation modifications mainly include Tip60 and p300. Tip60 is a transcriptional co‐activator, while ATF3 is a regulator of Tip60, and ATF3 acetylation enhances Tip60 activity and stability.^22^ Similarly, p300 is a transcriptional co‐activator and histone acetyltransferase, and the stimulation of glomerular thylakoid cells by subsoluble complement C5b‐9 complexes can upregulate p300 expression and then acetylate ATF3, thereby affecting ATF3 transcriptional activity without altering its sublocalization in the cell.^27^

##### Ubiquitination

Ubiquitination is the process of the specific modification of target proteins by ubiquitin molecules in the presence of ubiquitin‐activating enzymes (E1), ubiquitin‐binding enzymes (E2), and ubiquitin ligases (E3), leading to degradation of the target protein. Murine double minute 2 (MDM2) is an essential gene for the ubiquitination of ATF3. The ubiquitination of ATF3 mediated by the ubiquitin ligase MDM2 can lead to both the degradation of ATF3 and degradation of the oncogene p53 in cells, thus promoting tumor formation.^28^ In contrast, progesterone X receptors can block MDM2‐mediated ATF3 ubiquitination by targeting ATF3 lysine mutations, thereby increasing the stability of ATF3 and p53.^29^

##### Ubiquitin‐like modification

Both ubiquitin‐like and ubiquitin modifications are cascade reactions involving multiple enzymes. Ubiquitin‐like proteins are homologous and similar to ubiquitin proteins, but each ubiquitin‐like protein has its own specific biological function.^30^ Two representative examples are the small ubiquitin‐like modifier (SUMO) protein and neural precursor cell expressed developmentally downregulated (NEDD) 8 protein. Both SUMO and NEDD8 are small ubiquitin‐like peptides that also regulate intracellular processes by modifying specific proteins, and both require E1, E2, and E3, and hence the term “ubiquitin‐like proteins.”^31^ SUMO proteins function mainly in the nucleus and are involved in DNA replication, repair, and transcriptional regulation.^32^ The primary role of NEDDylation is to alter protein function, not to degrade it.^33^

ATF3 can be SUMOylated, and lysine 42 of ATF3 is the primary SUMO site. The SUMOylation of ATF3 does not directly interfere with the binding of ATF3 to DNA. However, how the SUMOylation of ATF3 alters its ability to recruit transcription factors remains unknown.^34^ In human umbilical vein endothelial cells, the SUMOylation of ATF3 promotes protein degradation caused by ATF3 ubiquitination, decreases ATF3 protein stability, and exacerbates angiotensin II‐induced inflammation and cellular dysfunction in endothelial cells. Additionally, SUMOylation controls the subcellular location of ATF3 in endothelial cells.^35^

In the process of NEDDylation, the protein is catalyzed by NEDD8 activase (E1), NEDD8 conjugase (E2), and NEDD8 ligase (E3).^33^ The most typical substrate of the NEDDylation pathway is Cullin‐RING E3 ubiquitin ligase (CRL). A study showed that the NEDD8‐activating enzyme inhibitor MLN4924 significantly enhances ATF3 expression at the protein and RNA levels. The stability of ATF3 was further determined using an actinomycin tracking assay after MLN4924 treatment, which revealed that the half‐life of ATF3 was not affected by MLN4924‐induced NEDD–CRL axis inactivation.^19^

Therefore, an in‐depth study of the various modalities of ATF3 posttranslational modifications is necessary to understand their mechanism of action.

## 3 ROLE OF ATF3 IN PULMONARY DISEASES

### 3.1 ALI/ARDS

ALI and ARDS are common diseases in the intensive care unit. One study reported that the 90‐day in‐hospital mortality rate of patients with moderate to severe ARDS was as high as 43%.^36^ Based on the available statistics, the causes of ARDS are complex, including severe pneumonia, sepsis, aspiration of gastric contents, and significant trauma. The pathogenesis of ARDS is mainly manifested as increased endothelial permeability, death and dysfunction of alveolar epithelial cells, loss of surfactant function, activation of the coagulation cascade, and activation of the pulmonary innate immune pathway.^37^ At present, the management of ARDS patients focuses on infection diagnosis and treatment, respiratory support, and fluid management. Moreover, individualized protocols and treatments developed based on these protocols often fail to meet clinical needs. Even so, the patient mortality rate remains high, so advancing precision medicine and gaining insights into the drivers of molecular heterogeneity may help identify new therapeutic targets.

**Table 1:** The role of ATF3 in inflammatory pulmonary diseases.

Diseases	Expression of ATF3	Signaling pathways	Roles of ATF3	References
ALI/ARDS	↑	LPS → ATF3 ↑ → TL1A ↓ /NF‐κB ↓ AUF1→Nrf2 ↑ /ATF3↓ ATF3 ↑ → DR5 ↑ /Bcl‐xL↓	Alleviate LPS/PA induced ALI Promote ferroptosis of alveolar epithelial cells Promote airway epithelium apoptosis	[38, 39, 40, 41]
PF	↑	CAE → ATF3 ↑ → PINK1↑Pirfenidone →ATF3 ↓/p‐Smad3↓	Initiate ERS Promote PF	[42, 43]
COPD	↑	CSE → ATF3 ↑ → MUC5AC↑ ATF3 ↑ →p NF‐ κB ↓	Promote COPD Alleviate COPD	[44, 45]

Abbreviations: ALI, acute lung injury; ARDS, acute respiratory distress syndrome; ATF3, activating transcription factor 3; AUF1, AU‐rich element RNA‐binding factor 1; Bcl‐Xl, B‐cell lymphoma‐extra large protein; CAE, citrus alkaline extract; COPD, chronic obstructive pulmonary disease; CSE, cigarette smoke extract; DR5, death receptor 5; ERS, endoplasmic reticulum stress; LPS, lipopolysaccharide; MUC5AC, mucin 5AC; NF‐κB, nuclear factor‐κB; Nrf2, nuclear factor erythroid 2‐related factor 2; pNF‐κB, phosphorylated NF‐κB; PA, *Pseudomonas aeruginosa*; PF, pulmonary fibrosis; PINK1, phosphatase and tensin homolog (PTEN)‐induced putative kinase 1; TL1A, tumor necrosis factor‐like ligand 1A.

**Figure 3:**
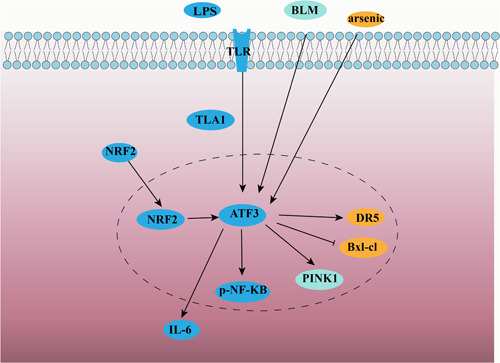
Signaling pathway of ATF3 in inflammatory pulmonary diseases.

#### 3.1.1 Effect of ATF3 in lipopolysaccharide (LPS)‐induced ALI

In a study exploring the expression profile of genes associated with lipopolysaccharide‐induced early ALI, protein–protein interaction network analysis showed that ATF3 was one of the most critical genes.^46^ Several studies have demonstrated that ATF3 plays an important role in the development of ALI induced by different factors.

A study found that compared to wild‐type mice, ATF3‐deficient mice were more likely to develop ALI with enhanced lung permeability, epithelial injury, and inflammation in response to LPS stimulation. ATF3 prevents over‐activation of the immune system by repressing the expression of pro‐inflammatory genes. Tumor necrosis factor (TNF)‐like cytokine 1A (TL1A), also called TNFSF15, is a member of the TNF family. Differential gene analysis showed that TL1A was highly expressed in LPS‐induced ATF3 knockout mice lung tissues, and that ATF3 downregulated TL1A expression in RAW264.7 cells and lung tissues.^38^ Like TL1A, the Toll‐like receptor (TLR) family plays a vital role in innate immunity. Upon recognition of their ligands, TLRs trigger complex intracellular signaling pathways and promote the expression of inflammatory cytokines, chemokines, and costimulatory molecules essential for activating adaptive immune responses.^47^ ATF3 is a negative regulator of the TLR4 signaling pathway.^48^

The central molecule in the TLR4 pathway is NF‐κB, which is translocated to the nucleus in cells to initiate the transcription of inflammatory mediators. The mammalian NF‐κB family consists of RelA (p65), c‐Rel, RelB, p50, and p52. ATF3 negatively regulates NF‐κB by interacting directly with p65.^49^ It was found in innate immunity that ATF3 attenuated inflammation by interacting with NF‐κB and inhibiting the LPS‐induced expression of the pro‐inflammatory cytokines IL‐6, IL‐12B, and TNF‐α in mice.^50^, ^51^ In contrast, ATF3 overexpression significantly increased p‐p65 (the phosphorylated form of p65) production in human bronchial epithelial cells in a particulate matter‐induced lung inflammation model, suggesting that ATF3 can act as an activator of the NF‐κB pathway in particulate matter‐induced inflammation.^8^ Thus, ATF3 acts as a critical immunomodulator against pathogen invasion, activating the immune response, and can enhance or suppress inflammation by regulating multiple target genes.^52^

#### 3.1.2 Effect of ATF3 in pneumonia‐associated ALI

*Pseudomonas aeruginosa* (PA) as an important opportunistic human pathogen can take part in the pathogenesis of pneumonia. Co‐immunoprecipitation in one study indicated that ATF3 protected mice against PA‐induced ALI in part by interacting with the LPS‐binding protein in lung tissue.^39^ Besides, enzyme‐linked immunosorbent assay results from BALF and peritoneal macrophages showed that ATF3 attenuates the release of inflammatory factors, such as TNF‐α, IL‐6, and IL‐1β. Furthermore, its protective effect may be related to the inhibition of NF‐κB activation, suggesting that the ATF3 gene may be a potential target gene for treating PA‐induced ALI.^39^ In pneumonia caused by *Staphylococcus aureus*, ATF3 regulates the expression of intracellular antimicrobial genes, actin cytoskeleton, and the cell migration of macrophages to ameliorate inflammation.^53^ In pneumonia caused by *Streptococcus pneumoniae*, ATF3 positively regulates the innate immunity during pneumococcal infection by enhancing TNF‐α, IL‐1β, and IFN‐γ expression and controlling bacterial clearance.^54^ ATF3 in macrophages promotes IL‐17A production in γδT cells for a rapid induction of the host defense during early *S. pneumoniae* infection.^55^ ATF3 overexpression after *S. pneumoniae* infection promotes cell proliferation in alveolar type II epithelial cells, which promotes lung epithelial recovery and improves lung function after injury.^56^

#### 3.1.3 Effect of ATF3 in sepsis‐associated ALI

Sepsis is a life‐threatening systemic condition and is the leading cause of ALI. Autophagy plays a vital role in regulating the pulmonary inflammatory response during sepsis. Studies have shown that ATF3 is a novel autophagy‐regulated gene. In autophagy‐deficient mice with the Atg4b gene knocked out, ATF3 is retained in the cytoplasm to prevent its binding to target genes.^57^ Ferroptosis is closely related to sepsis‐associated ALI. In a mouse model of sepsis induced by cecum ligation puncture (CLP), the inhibition of ferroptosis improved the survival rate of CLP mice and significantly alleviated lung tissue injuries. Further research revealed that AU‐rich element RNA binding factor 1 (AUF1) attenuated ferroptosis in alveolar epithelial cells in vitro by upregulating the expression of nuclear factor erythroid two related factors 2 (Nrf2) and downregulating the expression of ATF3.^40^ In addition to AUF1, the citrus flavonoid naringenin effectively attenuated lung tissue damage and reduced cytokine levels and leukocyte infiltration in septic mice.^58^ The above results suggest that ATF3 has a protective role in ALI.

#### 3.1.4 Effect of ATF3 on other types of ALI

The etiologies of ALI are complex. In addition to the above‐mentioned causes, ALI can also be induced by ischemia–reperfusion, ventilator application, and certain chemical substances. The mechanism of ALI caused by different etiologies is also different. In a rat model of lung transplantation injury, the expression of ATF3 increased significantly with the prolongation of the warm ischemic time (the time from the start of lung implantation to the release of the pulmonary artery clamp).^59^ In an ischemia–reperfusion‐induced lung injury model, it was found that carnosol (an antioxidant herbal compound) treatment may protect the lung from ischemia–reperfusion injury by regulating the ATF3–IL‐6 axis.^60^ Ventilator‐induced lung injury (VILI) can be triggered by mechanical injury, such as cyclic stretching. A microarray analysis of cyclic stretch response genes in Beas‐2B cells showed a significant enrichment of ATF3 in stretch cells. This study was validated in vivo, and the results were correlated and consistent with those from in vitro experiments. The research proves that ATF3 plays a protective role in VILI as an early stress gene.^61^ Nrf2 plays a protective role as a transcription factor mediated by antioxidant response elements. It was shown that ATF3 attenuates ventilator‐associated lung injury by preventing Nrf2 degradation.^62^ Nickel oxide nanoparticles (NiONPs) are commonly produced and used in industry, but are harmful to the lungs, and exposure to NiONPs can lead to apoptosis and ferroptosis in lung epithelial cells, ultimately leading to ALI.^63^ ATF3 expression was found to be upregulated in the lung tissue of mice and human lung epithelial cells after exposure to NiONPs. In another study, ATF3‐deficient BEAS‐2B cells were relatively resistant to apoptosis when exposed to arsenic, showing that ATF3 plays a facilitative role in arsenic‐induced apoptosis. ATF3 also prevented transcription of the death receptor 5 (DR5) and B‐cell lymphoma extra‐large protein (Bcl‐xL) by directly binding to the promoter DR5 and Bcl‐xL. ATF3 has the potential to serve as an early and sensitive biomarker for lung injury brought on by arsenic by acting as a proapoptotic protein in arsenic‐induced airway epithelium apoptosis.^41^

### 3.2 Pulmonary fibrosis

Pulmonary fibrosis (PF) is an end‐stage alteration of a large group of pulmonary diseases characterized by fibroblast proliferation and massive extracellular matrix aggregation with inflammatory damage and tissue structural destruction.^64^, ^65^ Its pathogenesis involves epithelial cell injury, aging, mitochondrial dysfunction, ER stress, and proteostasis imbalance.^66^ Although two antifibrotic drugs, nintedanib and pirfenidone, have been approved for the clinical treatment of PF, neither treatment is curative and lung transplantation is the only viable option for patients with PF.^67^, ^68^ However, the risks of surgery are high and new therapeutic approaches need to be explored to improve patient prognosis and enhance patients' long‐term quality of life.^69^

ATF3 may be a biomarker for PF.^70^ ATF3 was found to be significantly upregulated in lung tissues from mice with bleomycin‐induced pulmonary fibrosis and in patients with rheumatoid arthritis‐associated interstitial lung disease.^71^ In addition, the overexpression of ATF3 leads to the accumulation of depolarized mitochondria, increased mitochondrial ROS, and reduced cell viability, whereas the knockdown of ATF3 in type II lung epithelial cells was found to protect mice from bleomycin‐induced PF.^66^ Pirfenidone has a therapeutic effect on idiopathic pulmonary fibrosis (IPF). It also significantly reduced ATF3 expression and collagen accumulation in the bleomycin‐induced lung tissue of mice. Moreover, in one study where primary human lung fibroblasts (pHLFs) were treated in vitro with ATF3 shRNA‐expressing lentiviral vectors, it was found that the knockdown of ATF3 inhibited the production of p‐Smad3, an important mediator of pathological fibrosis. This suggests that pirfenidone inhibits myofibroblast differentiation by inhibiting the ATF3/Smad3 signaling pathway.^42^ Citrus alkaline extract (CAE) is derived from citrus pericarp and has antifibrotic properties. In one study, CAE inhibited the elevation of proteins downstream of ER stress induced by bleomycin or clathrin and activated the expression of ATF3. It also increased PTEN‐induced kinase 1 (PINK1) levels in type II alveolar epithelial cells in vivo and in vitro, suggesting that CAE may improve PF through the ATF3/PINK1 pathway.^43^ In studies of pulmonary inflammation and pulmonary fibrosis induced by inhaled silica particles, crystalline silica induced more intense stress‐related ATF3 gene expression and cytokine and chemokine secretion in primary human bronchial epithelial cells and mouse alveolar epithelial cells.^72^ However, a deeper understanding of the underlying mechanisms of disease development is a prerequisite for curing PF. Therefore, the mechanism of ATF3 in PF needs to be further investigated.

### 3.3 COPD

COPD is a clinical syndrome characterized by chronic respiratory symptoms, structural lung abnormalities (airway disease or emphysema), pulmonary dysfunction (mainly incomplete reversible airflow limitation), or any combination of the above. Chronic inflammation, protease‐antiprotease imbalance, ER stress, and oxidative stress are involved in the development of COPD.^73^, ^74^ The current treatments for COPD include the inhalation of β2‐adrenergic receptor agonists, glucocorticoids, and anticholinergics, but while these treatments help slow the progression of COPD, they have little effect in improving lung function and quality of life. Some of these drugs are also associated with toxic side effects, so there is an urgent need to find alternative therapies for COPD. Recent basic and clinical research has focused on the early pathophysiological changes in COPD to improve diagnosis and help identify patients most likely to benefit from early intervention.^75^ Although few new treatments have been approved for COPD in the last 5 years, new biomarker‐based strategies have made significant progress in targeting specific subgroups in existing therapies.^76^

The nature of COPD is as an inflammatory disease; especially, the acute exacerbation of COPD occurs with the aggregation of inflammatory cells and the massive release of inflammatory factors, which induce cupular cell hyperplasia of the airway epithelium and an increase in airway mucus secretion.^77^ The inflammatory factors stimulate the proliferation of smooth muscles and fibroblasts around the airway, leading to a remodeling of the small airways and aggravating the degree of airflow limitation. Excessive mucus secretion is one of the essential pathological features of COPD.^44^ Mucin 5AC (MUC5AC) is closely related to COPD mucus secretion. Studies have shown that cigarette smoke and cigarette smoke extract (CSE) stimulate ATF3 expression in mouse lung tissue and airway epithelial cells. ATF3 was found to be a positive regulator of CSE‐induced MUC5AC expression in vivo.^45^ It was suggested that ATF3 can promote mucus secretion and thus exert pro‐inflammatory effects in COPD models. However, the findings of another study were quite different. In animal experiments, ATF3 knockout mice showed significantly increased mucus production and increased peribronchial inflammatory cell infiltration after modeling compared to wild‐type mice, suggesting that ATF3 has an attenuating effect on COPD. In vitro experiments showed that ATF3 plays a negative regulatory role in mediating cigarette smoke‐induced inflammatory gene transcription, especially IL‐6 and IL‐8 expression, through the downregulation of pNF‐κB.^78^ The above results indicate that further studies on ATF3‐targeted therapies may be helpful for the treatment of cigarette smoke‐induced COPD.

## 4 CONCLUSION

ATF3 plays an important role in the development of multiple inflammatory pulmonary diseases (Table 1). This has been confirmed by a number of cell and animal experiments, but the specific mechanism remains to be explored (Figure 3). At present, studies on the effect of ATF3 in inflammatory pulmonary disease are limited to basic experiments, and there is a lack of in‐depth clinical research. In the future, new therapies and their delivery modes should be developed for ATF3, whether these ATF3‐targeted therapies need to be used in combination with specific drugs targeting other signaling pathways or alone.

## AUTHOR CONTRIBUTIONS

**Dandan Li**: Conceptualization (equal); investigation (lead); writing—original draft preparation (lead). **Juanjuan Jin**: Investigation (supporting); writing—original draft preparation (supporting). **Xue Dong**: Visualization (equal). **Chenyang Zhang**: Visualization (equal). **Jia Wang**: Conceptualization (equal); writing—review & editing (supporting). **Xianyao Wan**: Writing—review & editing (lead).

## CONFLICT OF INTEREST STATEMENT

The authors declare no conflict of interest.

## Data Availability

All data generated or analyzed during this study are included in this article.

## References

[1] Bhattacharya J , Matthay MA . Regulation and repair of the alveolar‐capillary barrier in acute lung injury. Annu Rev Physiol. 2013;75:593‐615. 23398155 10.1146/annurev-physiol-030212-183756

[2] Wang WJ , Ouyang C , Yu B , Chen C , Xu XF , Ye XQ . Role of hypoxia‑inducible factor‑2α in lung cancer (Review). Oncol Rep. 2021;45(5):57. 33760175 10.3892/or.2021.8008

[3] Alharbi KS , Fuloria NK , Fuloria S , et al. Nuclear factor‐kappa B and its role in inflammatory lung disease. Chem Biol Interact. 2021;345:109568. 34181887 10.1016/j.cbi.2021.109568

[4] Rohini M , Haritha Menon A , Selvamurugan N . Role of activating transcription factor 3 and its interacting proteins under physiological and pathological conditions. Int J Biiol Macromol. 2018;120(Pt A):310‐317. 10.1016/j.ijbiomac.2018.08.107 30144543

[5] Hai T , Hartman MG . The molecular biology and nomenclature of the activating transcription factor/cAMP responsive element binding family of transcription factors: activating transcription factor proteins and homeostasis. Gene. 2001;273(1):1‐11. 11483355 10.1016/s0378-1119(01)00551-0

[6] Eferl R , Wagner EF . AP‐1: a double‐edged sword in tumorigenesis. Nat Rev Cancer. 2003;3(11):859‐868. 14668816 10.1038/nrc1209

[7] Lu D , Chen J , Hai T . The regulation of ATF3 gene expression by mitogen‐activated protein kinases. Biochem J. 2007;401(2):559‐567. 17014422 10.1042/BJ20061081 PMC1820813

[8] Yan F , Wu Y , Liu H , Wu Y , Shen H , Li W . ATF3 is positively involved in particulate matter‐induced airway inflammation in vitro and in vivo. Toxicol Lett. 2018;287:113‐121. 29378244 10.1016/j.toxlet.2018.01.022

[9] Jiang HY , Wek SA , McGrath BC , et al. Activating transcription factor 3 is integral to the eukaryotic initiation factor 2 kinase stress response. Mol Cell Biol. 2004;24(3):1365‐1377. 14729979 10.1128/MCB.24.3.1365-1377.2004 PMC321431

[10] Zhou H , Li N , Yuan Y , et al. Activating transcription factor 3 in cardiovascular diseases: a potential therapeutic target. Basic Res Cardiol. 2018;113(5):37. 30094473 10.1007/s00395-018-0698-6

[11] Hai TW , Liu F , Coukos WJ , Green MR . Transcription factor ATF cDNA clones: an extensive family of leucine zipper proteins able to selectively form DNA‐binding heterodimers. Genes Dev. 1989;3(12B):2083‐2090. 2516827 10.1101/gad.3.12b.2083

[12] Hai T , Wolfgang CD , Marsee DK , Allen AE , Sivaprasad U . ATF3 and stress responses. Gene Expr. 1999;7(4‐6):321‐335. 10440233 PMC6174666

[13] Garces de Los Fayos Alonso I , Liang HC , Turner S , Lagger S , Merkel O , Kenner L . The role of Activator Protein‐1 (AP‐1) family members in CD30‐positive lymphomas. Cancers. 2018;10(4):93. 29597249 10.3390/cancers10040093 PMC5923348

[14] Ameri K , Hammond EM , Culmsee C , et al. Induction of activating transcription factor 3 by anoxia is independent of p53 and the hypoxic HIF signalling pathway. Oncogene. 2007;26(2):284‐289. 16847457 10.1038/sj.onc.1209781

[15] Chen SC , Liu YC , Shyu KG , Wang DL . Acute hypoxia to endothelial cells induces activating transcription factor 3 (ATF3) expression that is mediated via nitric oxide. Atherosclerosis. 2008;201(2):281‐288. 18377912 10.1016/j.atherosclerosis.2008.02.014

[16] Zhao J , Li X , Guo M , Yu J , Yan C . The common stress responsive transcription factor ATF3 binds genomic sites enriched with p300 and H3K27ac for transcriptional regulation. BMC Genomics. 2016;17:335. 27146783 10.1186/s12864-016-2664-8 PMC4857411

[17] Thompson MR , Xu D , Williams BRG . ATF3 transcription factor and its emerging roles in immunity and cancer. J Mol Med. 2009;87(11):1053‐1060. 19705082 10.1007/s00109-009-0520-x PMC2783469

[18] Avraham S , Korin B , Aviram S , Shechter D , Shaked Y , Aronheim A . ATF3 and JDP2 deficiency in cancer associated fibroblasts promotes tumor growth via SDF‐1 transcription. Oncogene. 2019;38(20):3812‐3823. 30670778 10.1038/s41388-019-0692-y PMC6756089

[19] Liang Y , Jiang Y , Jin X , et al. Neddylation inhibition activates the protective autophagy through NF‐κB‐catalase‐ATF3 axis in human esophageal cancer cells. Cell Commun Signal. 2020;18(1):72. 32398095 10.1186/s12964-020-00576-z PMC7218644

[20] Wang Z , He Y , Deng W , et al. Atf3 deficiency promotes genome instability and spontaneous tumorigenesis in mice. Oncogene. 2018;37(1):18‐27. 28869597 10.1038/onc.2017.310 PMC6179156

[21] Bathish B , Robertson H , Dillon JF , Dinkova‐Kostova AT , Hayes JD . Nonalcoholic steatohepatitis and mechanisms by which it is ameliorated by activation of the CNC‐bZIP transcription factor Nrf2. Free Radic Biol Med. 2022;188:221‐261. 35728768 10.1016/j.freeradbiomed.2022.06.226

[22] Cui H , Guo M , Xu D , et al. The stress‐responsive gene ATF3 regulates the histone acetyltransferase Tip60. Nat Commun. 2015;6:6752. 25865756 10.1038/ncomms7752 PMC4407828

[23] Duncan RM , Reyes L , Moats K , et al. ATF3 coordinates antitumor synergy between epigenetic drugs and protein disulfide isomerase inhibitors. Cancer Res. 2020;80(16):3279‐3291. 32561529 10.1158/0008-5472.CAN-19-4046 PMC7442646

[24] Inuzuka H , Gao D , Finley LWS , et al. Acetylation‐dependent regulation of Skp2 function. Cell. 2012;150(1):179‐193. 22770219 10.1016/j.cell.2012.05.038 PMC3595190

[25] Shen F , Boccuto L , Pauly R , Srikanth S , Chandrasekaran S . Genome‐scale network model of metabolism and histone acetylation reveals metabolic dependencies of histone deacetylase inhibitors. Genome Biol. 2019;20(1):49. 30823893 10.1186/s13059-019-1661-z PMC6397465

[26] Gao T , Yang C , Zheng YG . Comparative studies of thiol‐sensitive fluorogenic probes for HAT assays. Anal Bioanal Chem. 2013;405(4):1361‐1371. 23138472 10.1007/s00216-012-6522-5 PMC3548978

[27] He F , Zhou M , Yu T , et al. Sublytic C5b‐9 triggers glomerular mesangial cell apoptosis in rat Thy‐1 nephritis via Gadd45 activation mediated by Egr‐1 and p300‐dependent ATF3 acetylation. J Mol Cell Biol. 2016;8(6):477‐491. 27190312 10.1093/jmcb/mjw021

[28] Li X , Guo M , Cai L , et al. Competitive ubiquitination activates the tumor suppressor p53. Cell Death Differ. 2020;27(6):1807‐1818. 31796886 10.1038/s41418-019-0463-x PMC7244561

[29] Niu X , Cui H , Gu X , et al. Nuclear receptor PXR confers irradiation resistance by promoting DNA damage response through stabilization of ATF3. Front Oncol. 2022;12:837980. 35372071 10.3389/fonc.2022.837980 PMC8965888

[30] Swatek KN , Komander D . Ubiquitin modifications. Cell Res. 2016;26(4):399‐422. 27012465 10.1038/cr.2016.39 PMC4822133

[31] Ball KA , Johnson JR , Lewinski MK , et al. Non‐degradative ubiquitination of protein kinases. PLoS Comput Biol. 2016;12(6):e1004898. 27253329 10.1371/journal.pcbi.1004898 PMC4890936

[32] Boulanger M , Chakraborty M , Tempé D , Piechaczyk M , Bossis G . SUMO and transcriptional regulation: the lessons of large‐scale proteomic, modifomic and genomic studies. Molecules. 2021;26(4):828. 33562565 10.3390/molecules26040828 PMC7915335

[33] Zhao Y , Morgan MA , Sun Y . Targeting neddylation pathways to inactivate cullin‐RING ligases for anticancer therapy. Antioxid Redox Signal. 2014;21(17):2383‐2400. 24410571 10.1089/ars.2013.5795 PMC4241876

[34] Wang CM , Yang WH . Loss of SUMOylation on ATF3 inhibits proliferation of prostate cancer cells by modulating CCND1/2 activity. Int J Mol Sci. 2013;14(4):8367‐8380. 23591848 10.3390/ijms14048367 PMC3645748

[35] Zhang ZB , Ruan CC , Chen DR , Zhang K , Yan C , Gao PJ . Activating transcription factor 3 SUMOylation is involved in angiotensin II‐induced endothelial cell inflammation and dysfunction. J Mol Cell Cardiol. 2016;92:149‐157. 26850942 10.1016/j.yjmcc.2016.02.001

[36] National Heart L . Early neuromuscular blockade in the acute respiratory distress syndrome. N Engl J Med. 2019;380(21):1997‐2008. 31112383 10.1056/NEJMoa1901686 PMC6741345

[37] Meyer NJ , Gattinoni L , Calfee CS . Acute respiratory distress syndrome. Lancet. 2021;398(10300):622‐637. 34217425 10.1016/S0140-6736(21)00439-6 PMC8248927

[38] Qian L , Zhao Y , Guo L , Li S , Wu X . Activating transcription factor 3 (ATF3) protects against lipopolysaccharide‐induced acute lung injury via inhibiting the expression of TL1A. J Cell Physiol. 2017;232(12):3727‐3734. 28177121 10.1002/jcp.25849

[39] Zhao Y , Wu X , Qian L , Guo L , Liao J , Wu X . Activating transcription factor 3 protects mice against *Pseudomonas aeruginosa*‐induced acute lung injury by interacting with lipopolysaccharide binding protein. Mol Immunol. 2017;90:27‐32. 28662411 10.1016/j.molimm.2017.06.037

[40] Wang Y , Chen D , Xie H , et al. AUF1 protects against ferroptosis to alleviate sepsis‐induced acute lung injury by regulating NRF2 and ATF3. Cell Mol Life Sci. 2022;79(5):228. 35391558 10.1007/s00018-022-04248-8 PMC11072094

[41] Shi Q , Hu B , Yang C , Zhao L , Wu J , Qi N . ATF3 promotes arsenic‐induced apoptosis and oppositely regulates DR5 and Bcl‐xL expression in human bronchial epithelial cells. Int J Mol Sci. 2021;22:4223. 33921748 10.3390/ijms22084223 PMC8072958

[42] Wu C , Lin H , Zhang X . Inhibitory effects of pirfenidone on fibroblast to myofibroblast transition in rheumatoid arthritis‐associated interstitial lung disease via the downregulation of activating transcription factor 3 (ATF3). Int Immunopharmacol. 2019;74:105700. 31228816 10.1016/j.intimp.2019.105700

[43] Wang Z , Feng F , He H , et al. Citrus alkaline extracts prevent endoplasmic reticulum stress in type II alveolar epithelial cells to ameliorate pulmonary fibrosis via the ATF3/PINK1 pathway. Phytomedicine. 2021;89:153599. 34260993 10.1016/j.phymed.2021.153599

[44] Radicioni G , Ceppe A , Ford AA , et al. Airway mucin MUC5AC and MUC5B concentrations and the initiation and progression of chronic obstructive pulmonary disease: an analysis of the SPIROMICS cohort. Lancet Respir Med. 2021;9(11):1241‐1254. 34058148 10.1016/S2213-2600(21)00079-5 PMC8570975

[45] Wu Y , Wu Y , Zhang C , et al. Activating transcription factor 3 is essential for cigarette smoke‐induced mucin expression via interaction with activator Protein‐1. Am J Pathol. 2017;187(2):280‐291. 27912076 10.1016/j.ajpath.2016.10.012

[46] Luo D , Liu F , Zhang J , et al. Comprehensive analysis of LncRNA‐mRNA expression profiles and the ceRNA network associated with pyroptosis in LPS‐induced acute lung injury. J Inflamm Res. 2021;14:413‐428. 33628043 10.2147/JIR.S297081 PMC7898231

[47] Kawai T , Akira S . Toll‐like receptors and their crosstalk with other innate receptors in infection and immunity. Immunity. 2011;34(5):637‐650. 21616434 10.1016/j.immuni.2011.05.006

[48] Nguyen CT , Kim EH , Luong TT , Pyo S , Rhee DK . TLR4 mediates pneumolysin‐induced ATF3 expression through the JNK/p38 pathway in *Streptococcus pneumoniae*‐infected RAW 264.7 cells. Mol Cells. 2015;38(1):58‐64. 25518930 10.14348/molcells.2015.2231 PMC4314132

[49] Kwon JW , Kwon HK , Shin HJ , Choi YM , Anwar MA , Choi S . Activating transcription factor 3 represses inflammatory responses by binding to the p65 subunit of NF‐κB. Sci Rep. 2015;5:14470. 26412238 10.1038/srep14470 PMC4585983

[50] Gilchrist M , Thorsson V , Li B , et al. Systems biology approaches identify ATF3 as a negative regulator of Toll‐like receptor 4. Nature. 2006;441(7090):173‐178. 16688168 10.1038/nature04768

[51] Ding DX , Tian FF , Guo JL , et al. Dynamic expression patterns of ATF3 and p53 in the hippocampus of a pentylenetetrazole‐induced kindling model. Mol Med Rep. 2014;10(2):645‐651. 24859284 10.3892/mmr.2014.2256 PMC4094765

[52] Hai T , Wolford CC , Chang YS . ATF3, a hub of the cellular adaptive‐response network, in the pathogenesis of diseases: is modulation of inflammation a unifying component? Gene Expr. 2010;15(1):1‐11. 21061913 10.3727/105221610x12819686555015 PMC6043823

[53] Du Y , Ma Z , Zheng J , et al. ATF3 positively regulates antibacterial immunity by modulating macrophage killing and migration functions. Front Immunol. 2022;13:839502. 35370996 10.3389/fimmu.2022.839502 PMC8965742

[54] Nguyen CT , Kim EH , Luong TT , Pyo S , Rhee DK . ATF3 confers resistance to pneumococcal infection through positive regulation of cytokine production. J Infect Dis. 2014;210(11):1745‐1754. 24951825 10.1093/infdis/jiu352

[55] Lee S , Kim GL , Kim NY , Kim SJ , Ghosh P , Rhee DK . ATF3 stimulates IL‐17A by regulating intracellular Ca2+/ROS‐dependent IL‐1β activation during *Streptococcus pneumoniae* infection. Front Immunol. 2018;9:1954. 30214444 10.3389/fimmu.2018.01954 PMC6125349

[56] Ali M , LaCanna R , Lian Z , et al. Transcriptional responses to injury of regenerative lung alveolar epithelium. iScience. 2022;25(8):104843. 35996586 10.1016/j.isci.2022.104843 PMC9391595

[57] Aguirre A , López‐Alonso I , González‐López A , et al. Defective autophagy impairs ATF3 activity and worsens lung injury during endotoxemia. J Mol Med. 2014;92(6):665‐676. 24535031 10.1007/s00109-014-1132-7

[58] Liu X , Wang N , Fan S , et al. The citrus flavonoid naringenin confers protection in a murine endotoxaemia model through AMPK‐ATF3‐dependent negative regulation of the TLR4 signalling pathway. Sci Rep. 2016;6:39735. 28004841 10.1038/srep39735 PMC5177915

[59] Yamamoto S , Yamane M , Yoshida O , et al. Activations of mitogen‐activated protein kinases and regulation of their downstream molecules after rat lung transplantation from donors after cardiac death. Transplant Proc. 2011;43(10):3628‐3633. 22172817 10.1016/j.transproceed.2011.09.075

[60] Momozane T , Kawamura T , Itoh Y , et al. Carnosol suppresses interleukin‐6 production in mouse lungs injured by ischemia‐reperfusion operation and in RAW264.7 macrophages treated with lipopolysaccharide. Biochem Cell Biol. 2018;96(6):769‐776. 29958095 10.1139/bcb-2017-0339

[61] Akram A , Han B , Masoom H , et al. Activating transcription factor 3 confers protection against ventilator‐induced lung injury. Am J Respir Crit Care Med. 2010;182(4):489‐500. 20413626 10.1164/rccm.200906-0925OC PMC2937241

[62] Shan Y , Akram A , Amatullah H , et al. ATF3 protects pulmonary resident cells from acute and ventilator‐induced lung injury by preventing Nrf2 degradation. Antioxid Redox Signal. 2015;22(8):651‐668. 25401197 10.1089/ars.2014.5987 PMC4346377

[63] Liu F , Cheng X , Wu S , et al. Nickel oxide nanoparticles induce apoptosis and ferroptosis in airway epithelial cells via ATF3. Environ Toxicol. 2022;37(5):1093‐1103. 35061333 10.1002/tox.23467

[64] King, Jr. TE , Pardo A , Selman M . Idiopathic pulmonary fibrosis. Lancet. 2011;378(9807):1949‐1961. 21719092 10.1016/S0140-6736(11)60052-4

[65] Fernandez IE , Eickelberg O . New cellular and molecular mechanisms of lung injury and fibrosis in idiopathic pulmonary fibrosis. Lancet. 2012;380(9842):680‐688. 22901889 10.1016/S0140-6736(12)61144-1

[66] Moss BJ , Ryter SW , Rosas IO . Pathogenic mechanisms underlying idiopathic pulmonary fibrosis. Annu Rev Pathol Mech Dis. 2022;17:515‐546. 10.1146/annurev-pathol-042320-030240 34813355

[67] Wilson‐Smith AR , Kim YS , Evans GE , Yan TD . Single versus double lung transplantation for fibrotic disease‐systematic review. Ann Cardiothorac Surg. 2020;9(1):10‐19. 32175235 10.21037/acs.2019.12.04 PMC7049556

[68] Loomis‐King H , Flaherty KR , Moore BB . Pathogenesis, current treatments and future directions for idiopathic pulmonary fibrosis. Curr Opin Pharmacol. 2013;13(3):377‐385. 23602652 10.1016/j.coph.2013.03.015 PMC3686907

[69] Chambers DC , Cherikh WS , Harhay MO , et al. The International Thoracic Organ Transplant Registry of the International Society for Heart and Lung Transplantation: thirty‐sixth adult lung and heart‐lung transplantation Report‐2019; Focus theme: Donor and recipient size match. J Heart Lung Transplant. 2019;38(10):1042‐1055. 31548030 10.1016/j.healun.2019.08.001 PMC6816340

[70] Maghsoudloo M , Azimzadeh Jamalkandi S , Najafi A , Masoudi‐Nejad A . Identification of biomarkers in common chronic lung diseases by co‐expression networks and drug‐target interactions analysis. Mol Med. 2020;26(1):9. 31952466 10.1186/s10020-019-0135-9 PMC6969427

[71] Raghu G , Chen SY , Hou Q , Yeh WS , Collard HR . Incidence and prevalence of idiopathic pulmonary fibrosis in US adults 18‐64 years old. Eur Respir J. 2016;48(1):179‐186. 27126689 10.1183/13993003.01653-2015

[72] Perkins TN , Shukla A , Peeters PM , et al. Differences in gene expression and cytokine production by crystalline vs. amorphous silica in human lung epithelial cells. Part Fibre Toxicol. 2012;9(1):6. 22300531 10.1186/1743-8977-9-6 PMC3337246

[73] Agustí A , Hogg JC . Update on the pathogenesis of chronic obstructive pulmonary disease. N Engl J Med. 2019;381(13):1248‐1256. 31553836 10.1056/NEJMra1900475

[74] Celli BR , Wedzicha JA . Update on clinical aspects of chronic obstructive pulmonary disease. N Engl J Med. 2019;381(13):1257‐1266. 31553837 10.1056/NEJMra1900500

[75] Ferrera MC , Labaki WW , Han MK . Advances in chronic obstructive pulmonary disease. Annu Rev Med. 2021;72:119‐134. 33502902 10.1146/annurev-med-080919-112707 PMC8011854

[76] Christenson SA , Smith BM , Bafadhel M , Putcha N . Chronic obstructive pulmonary disease. Lancet. 2022;399(10342):2227‐2242. 35533707 10.1016/S0140-6736(22)00470-6

[77] Wang C , Zhou J , Wang J , et al. Progress in the mechanism and targeted drug therapy for COPD. Signal Transduct Target Ther. 2020;5(1):248. 33110061 10.1038/s41392-020-00345-x PMC7588592

[78] Wu Y , Cao C , Wu Y , et al. Activating transcription factor 3 represses cigarette smoke‐induced IL6 and IL8 expression via suppressing NF‐κB activation. Toxicol Lett. 2017;270:17‐24. 28185985 10.1016/j.toxlet.2017.02.002

